# A century of cardiac rehabilitation research: Bibliometric review of publication history, keyword trends, and citations

**DOI:** 10.1038/s44325-025-00062-w

**Published:** 2025-06-26

**Authors:** Deborah Manandi, Karice Hyun, Dion Candelaria, Matthew Hollings, Qiang Tu, Sarah Gauci, Adrienne O’Neil, Georgia K. Chaseling, Ling Zhang, Tom Briffa, Sherry L. Grace, Robyn Gallagher, Julie Redfern

**Affiliations:** 1 Susan Wakil School of Nursing and Midwifery, Faculty of Medicine and Health, University of Sydney, Sydney, NSW Australia; 2 Sydney School of Health Sciences, Faculty of Medicine and Health, University of Sydney, Sydney, NSW Australia; 3 Department of Cardiology, Concord Repatriation General Hospital, ANZAC Research Institute, Sydney, NSW Australia; 4 IMPACT—The Institute for Mental and Physical Health and Clinical Translation, Food & Mood Centre, School of Medicine, Deakin University, Geelong, VIC Australia; 5 School of Population and Global Health, University of Western Australia, Perth, WA Australia; 6 School of Kinesiology and Health Science, York University, Toronto, ON Canada; 7 KITE Research Institute, Toronto Rehabilitation & Peter Munk Cardiac Centre, University Health Network, University of Toronto, Toronto, ON Canada; 8 Institute for Evidence-Based Healthcare, Bond University, Gold Coast, QLD Australia; 9 The George Institute for Global Health, University of New South Wales, Sydney, NSW Australia

**Keywords:** Cardiology, Health care

## Abstract

Research into cardiac rehabilitation (CR), a key model for secondary prevention of cardiovascular disease, has evolved since first described in 1927. This review aimed to explore this evolution by identifying CR-related publications from the Web of Science Core Collection and summarizing CR research publication history, trends in publication keywords, and citations over time. A total of 8729 CR publications appeared across 1441 journals (median impact factor: 2.6) and were cited 315,819 times, with over 85% (7455/8729) published in the past two decades. These publications involved contributions from 26,909 authors across 120 countries, despite disproportionate domination by high-income countries. Publication keywords have consistently focused on exercise but have evolved from evaluating clinical events, quality-of-life, and return-to-work outcomes to improving accessibility using digital interventions. However, a broader focus on other cardiovascular risk factors, comorbidities, and various research designs may be needed to modernize CR, particularly in lower-income countries.

## Introduction

Cardiovascular disease (CVD) research has led to advancements in the prevention and management of CVD^1–3^. While there has been a steep global decline in CVD mortality−from 355 deaths per 100,000 people in 1990 to 240 in 2019−the prevalence of CVD has nearly doubled from 271 to 523 million, and years lived with CVD disability have also nearly doubled from 18 to 34 million^1,4^. This increased prevalence indicates that although fewer people are dying from CVD, many are living longer with associated complications, requiring lifelong management^5^.

Scientific publications have accompanied this progress in CVD prognosis and outcomes^6^. Early publications addressed issues such as the reintegration of “cardiacs” into the workforce^7^. The development of catheterization procedures in 1944, followed by the integration of imaging techniques starting in 1953, revolutionized the diagnosis of CVD^8,9^. In the 1950s, the Framingham Heart Study identified risk factors such as hypertension, dyslipidemia, diabetes, and obesity^10–12^. The introduction of selective coronary angiography and bypass procedures in the 1960s, then stents and catheter-based interventions in the late 1970s, transformed treatment approaches for cardiac emergencies^13–16^. Alongside these developments and spurred on by the greater survival of initial CVD events, there was the development of cardiac rehabilitation^6^. This model of care initially focused on mobilization, subsequently exercise training, and as evidence evolved, expanded outpatient delivery, including disease education and psychosocial support for comprehensive secondary prevention in the 1970s^6^. More recently, the integration of technology and digital platforms gained momentum following the COVID-19 pandemic in 2020, revolutionizing remote CVD care^17^.

Research demonstrates the clear benefits of cardiac rehabilitation, including significant improvements in quality-of-life, reductions in recurrent CVD events, a 23% reduction in CVD rehospitalizations, and a 26% reduction in CVD mortality^18^. Despite these clear benefits, participation rates remain low, ranging from 7% to 35%, with even lower rates in lower-resource settings where the burden of CVD is increasing^19–24^. Furthermore, the structure of cardiac rehabilitation programs has remained largely unchanged^6,25^, raising questions about how research has influenced program delivery innovation and advancement beyond effectiveness. With longer life expectancy, the global burden of CVD will persist, imposing challenges for health systems. All countries will be affected by the future burden of CVD unless cardiac rehabilitation is modernized to adapt to the competing health resources and changing CVD landscape^4–6^.

To address these challenges and guide the modernization of cardiac rehabilitation, it is important to explore how research in the field of cardiac rehabilitation has evolved over time. Bibliometric analysis can provide an overview of this evolution by summarizing publication history, geographic contributions, citation patterns, and publication keywords^22,26–28^. A bibliometric review also complements other study designs by capturing long-term trends and identifying underrepresented research focus areas that may not be addressed in individual studies, including systematic review, and by guiding future research within the changing context of CVD and secondary prevention. This bibliometric review aimed to summarize cardiac rehabilitation research publication history (journal field and quality, number, country, author collaboration), publication keywords, and citations over time to understand developments and apprise future research needs.

## Results

### Publication history: Journal field and quality

**Fig. 1:**
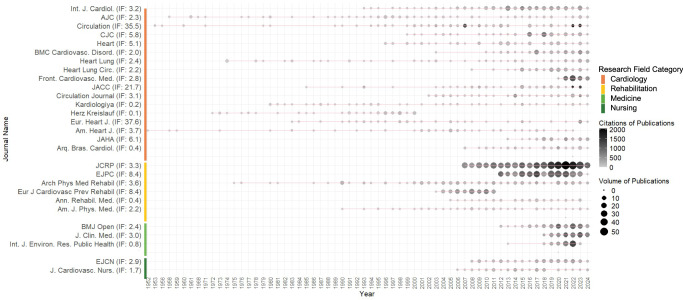
Journal name, as well as annual number and citations of cardiac rehabilitation publications by full calendar year, ranked by the journal’s research field category and number of publications. The figure presents journal names alongside the number and citation count of cardiac rehabilitation publications per year, ranked by research field category and total number of publications. *Int. J. Cardiol.International Journal of Cardiology*, *AJCAmerican Journal of Cardiology*, *CJCCanadian Journal of Cardiology*, *BMC Cardiovasc. Disord.BMC Cardiovascular Disorders*, *Heart LungHeart & Lung*, *Heart Lung Circ.Heart, Lung and Circulation*, *Front. Cardiovasc. Med.Frontiers in Cardiovascular Medicine*, *JACCJournal of the American College of Cardiology*, *Eur. Heart J.European Heart Journal*, *Am. Heart J.American Heart Journal*, *JAHAJournal of the American Heart Association*, *Arq. Bras. Cardiol.Arquivos Brasileiros de Cardiologia*, *JCRPJournal of Cardiopulmonary Rehabilitation and Prevention*, *EJPCEuropean Journal of Preventive Cardiology*, *Arch. Phys. Med. Rehabil.Archives of Physical Medicine and Rehabilitation*, *Eur. J. Cardiovasc. Prev. Rehabil.European Journal of Cardiovascular Prevention & Rehabilitation*, *Ann. Rehabil. Med.Annals of Rehabilitation Medicine*, *Am. J. Phys. Med.American Journal of Physical Medicine & Rehabilitation*, *J. Clin. Med.Journal of Clinical Medicine*, *Int. J. Environ. Res. Public HealthInternational Journal of Environmental Research and Public Health*, *EJCNEuropean Journal of Cardiovascular Nursing*, *J. Cardiovasc. Nurs.Journal of Cardiovascular Nursing*.

A total of 1441 journals published cardiac rehabilitation research. Most (*n* = 1257, 87%) of these journals were based in high-income countries (highest number based in the United States, the United Kingdom and Germany); 113 (7.8%) were based in upper-middle-income countries (highest number based in Brazil, Russia and China); 62 (4.3%) were based in lower-middle-income countries (highest number based in India, Iran and Pakistan); while 2 (0.1%) were based in low-income countries (Ethiopia and Malawi). The highest number of cardiac rehabilitation publications were in the *Journal of Cardiopulmonary Rehabilitation and Prevention* (*n* = 571; 6.5%), followed by the *European Journal of Preventive Cardiology* (*n* = 302, 3.5%) and the *International Journal of Cardiology* (*n* = 168, 1.9%; Fig. 1). Out of the journals with an impact factor in 2023 (*n* = 703, 49%), the median (interquartile range, IQR) impact factor was 2.6 (1.7–3.7).

There were 28 journals that published at least 50 cardiac rehabilitation-related publications. Of these, 17 (61%) were in the subspecialty field of cardiology, 6 (21%) in rehabilitation or physiatry, 3 (11%) in medicine and 2 (7.1%) in nursing, with an increasing number of publications in the recent years in nursing (Fig. 1). Under the subspecialty of cardiology, the two most cardiac rehabilitation-published journals are: (1) the *International Journal of Cardiology* (Field-weighted citation impact [FWCI] in 2023: 1.01, H-index: 155), and (2) the *American Journal of Cardiology* (FWCI in 2023: 0.59, H-index: 243). Under the subspecialty of rehabilitation, the two most cardiac rehabilitation-published journals are: (1) the *Journal of Cardiopulmonary Rehabilitation and Prevention* (FWCI in 2023: 0.38, H-index: 80), and (2) the *European Journal of Preventive Cardiology* (FWCI in 2023: 1.92, H-index: 130). Under the specialty of medicine, the two most cardiac rehabilitation-published journals are: (1) the *BMJ Open* (FWCI in 2023: 0.94, H-index: 176), and (2) the *Journal of Clinical Medicin*e (FWCI in 2023: 1.12, H-index: 132). Meanwhile, under the subspecialty of nursing, the two most cardiac rehabilitation-published journals are: (1) the *European Journal of Cardiovascular Nursing* (FWCI in 2023: 2.74, H-index: 66), and (2) the *Journal of Cardiovascular Nursing* (FWCI in 2023: 0.90, H-index: 73).

### Publication history: Publication numbers, country, and author collaboration

**Fig. 2:**
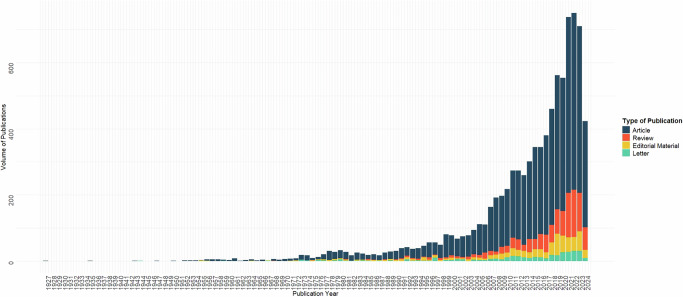
Annual number of cardiac rehabilitation publications (i.e., original research articles, reviews, editorial materials, and letters) by full calendar year from 1927 to 2023. The figure presents the number of cardiac rehabilitation publications per year, grouped by publication type from 1927 to 2023.

Up to the search date (August 12th, 2024), a total of 8729 publications were identified, comprising 72% (*n* = 6629) full original research articles, 13% (*n* = 1106) reviews, 7.4% (*n* = 651) editorial materials, and 3.9% (*n* = 343) letters. Cardiac rehabilitation publications were infrequent between 1927 and 1950, before appearing annually from 1952 onwards (Fig. 2).

The number of publications has grown substantially, particularly over the past two decades, when 85% (7455/8729) of all publications were published. For the first 70 years, the annual number of publications increased gradually (Fig. 2). The growth became exponential in the early 2000s, peaking at over 700 publications annually by 2022, despite slight declines in 2013, 2020, and 2023 (Fig. 2).

**Fig. 3:**
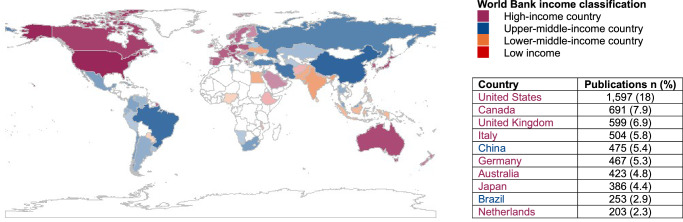
Number of cardiac rehabilitation publications (i.e., original research articles, reviews, editorial materials, and letters) by country and World Bank income classification of the country of the corresponding author. The figure presents the number of cardiac rehabilitation publications by the corresponding author’s country, grouped by World Bank income classification and shaded by the number of publications. Note: Shading represents the number of publications in descending gradient within each income classification.

Collectively, cardiac rehabilitation publications involved 26,909 unique authors across 2670 institutional affiliations in 120 of the ~194 countries globally (Fig. 3). However, publications from authors in lower-income countries were limited, with particularly low representation from the African region. Most publications (*n* = 7823, 90%) involved collaboration among a median (IQR) of 5 (3–8) authors, with 17% (*n* = 1460) including multi-national collaboration.

Corresponding authors in high-income countries published 7.7 times more than those in upper-middle-income, and 22 times more than those in lower-middle-income countries. The highest number of publications was from corresponding authors in the United States (*n* = 1597; 18%) followed by Canada (*n* = 691, 7.9%) and the United Kingdom (*n* = 599, 6.9%). The 10 most-published corresponding authors’ countries with more than 200 publications, along with their income classifications, across all time, are shown in Fig. 3. The number of publications from corresponding authors in upper-middle-income countries has increased significantly since 2020. Early publications in upper-middle-income countries were from Argentina in 1978, Russia in 1992, and Azerbaijan in 1994. Over 58% (553/959) of publications in upper-middle-income countries were published between 2020 and the search date (August 12th, 2024). Similarly, in lower-middle-income countries, early publications were from Iran in 2003, Egypt in 2007, and Jordan in 2009. Over 51% (175/341) of publications in lower-middle-income countries were published during the same period. Only three publications had corresponding authors from low-income countries (Malawi in 2021, Ethiopia in 2022, and Afghanistan in 2023).

### Publication keyword

**Fig. 4:**
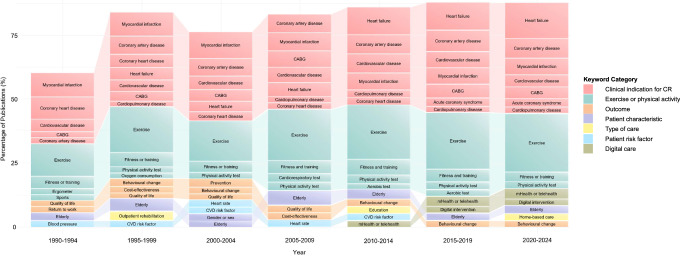
Percentage of publications using the top 15 most frequently used keywords per 5-year period, in descending order by keyword category and within each category. The figure presents the percentage of cardiac rehabilitation publications using the 15 most frequently used keywords per 5-year period, grouped and ordered by keyword category. CABG coronary artery bypass graft, CVD cardiovascular disease, mHealth mobile health, 6-min walk test six-minute walk test, CR cardiac rehabilitation. Note: The Aerobic test evaluates an individual’s capacity to consume and utilize oxygen during sustained physical activity, serving as an indicator of their endurance. The cardiorespiratory test evaluates an individual’s efficiency in delivering oxygen to active muscle and expelling carbon dioxide during physical activity, serving as an indicator of their integrated cardiovascular and respiratory fitness. Physical activity test tracks an individual’s physical activity patterns, habits, and intensity using tools such as questionnaires.

From 1990 onwards, when author-assigned keywords became available, cardiac rehabilitation publications have used a total of 539 unique keywords. Across each 5-year period, notable homogeneity was observed, with more than 50% of publications consistently using the same 15 keywords (Fig. 4). These keywords primarily related to clinical indications for cardiac rehabilitation, exercise or physical activity, outcomes, patient characteristics, type of care, patient risk factors, and digital care, as visualized in Fig. 4. The most frequently used keywords related to research design, were randomized controlled trials, followed by qualitative studies, systematic reviews, surveys and quality improvement studies.

Keyword related to exercise have remained a consistent focus, with around 20% of publications using the keyword in each 5-year period (Fig. 4). The most frequently used keyword related to clinical indications for cardiac rehabilitation has shifted over time, with myocardial infarction being the primary focus in earlier publications, whereas since 2010, heart failure has become a more commonly used keyword (Fig. 4). Between 1995 and 2009, there was an increased emphasis on keywords related to cardiac rehabilitation patient outcomes or efficacy, such as quality-of-life, return-to-work, cost-effectiveness, and adherence−either to the program or to heart-health behaviors (Fig. 4). Since 2010, keywords have increasingly focused on new delivery models, such as patient education, digital interventions and home-based care (Fig. 4).

In contrast, less frequently used keywords related to patient risk factors included depression, anxiety, diabetes, nutrition, and obesity. Similarly, keywords related to patient sub-groups, such as the elderly, or other outcomes such as cardioprotective medication adherence or patient satisfaction, have remained underrepresented across the history of this literature.

### Citations

Cardiac rehabilitation publications have received a total of 315,819 citations across all time up to the search date (August 12th, 2024), with each publication being cited a median (IQR) of 31 (19–44) times. However, corresponding authors in high-income countries were cited 7.7 times more than those in upper-middle-income countries, and 27 times more than those in lower-middle-income countries.

**Fig. 5:**
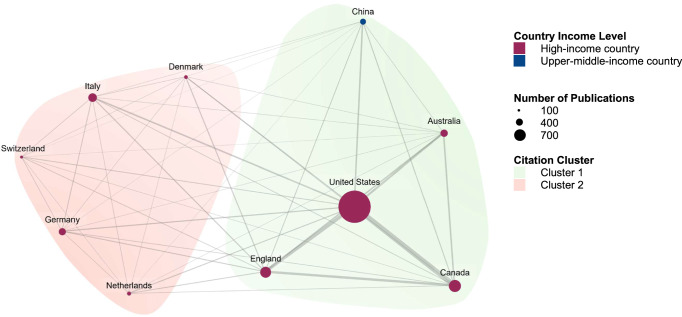
Citation patterns of cardiac rehabilitation publications by country and World Bank income classification of the country of the corresponding author. The figure presents citation patterns between countries based on the corresponding author’s affiliation, grouped by World Bank income classification. Note: Lines between dots represent citation links.

Among the 10 most-cited countries, the United States of America, Canada and England formed a citation cluster, with the strongest link between the United States and Canada, followed by the United States and England, and then Canada with England (Fig. 5). This cluster also included Australia and China (Fig. 5). Whereas several European countries, specifically Denmark, Germany, Italy, Netherlands and Switzerland, formed a separate citation cluster (Fig. 5).

## Discussion

The field of cardiac rehabilitation research, comprising over 8000 publications and over 300,000 citations, has been comprehensively summarized in the current review, detailing its history, publication keywords and citations from inception to the present^22,26–28^. Publications appeared across over 14,000 journals with a median impact factor of 2.6 in 2023, most frequently in two subspecialty journals: the *Journal of Cardiopulmonary Rehabilitation and Prevention* and the *European Journal of Preventive Cardiology*. Cardiac rehabilitation-related publications first appeared infrequently between 1927 and 1950, became consistent, and experienced substantial growth from 2004 onwards, reaching over 700 publications annually from 2021 onwards, and marking nearly a century of progress. While a median of five authors contributed to each publication, the over 26,000 authors involved in cardiac rehabilitation research across all time represented nearly two-thirds of countries globally. However, high-income countries have disproportionately dominated the number and citation of publications compared to lower-income countries. The field has consistently focused on exercise, progressing to establish efficacy, and more recently has expanded to address additional clinical indications and integrate technology-based delivery models^17,29,30^.

The substantial growth in the cardiac rehabilitation research over the past 20 years mirrors similar trends seen across subspecialty fields within cardiology, such as CVD risk factors; specialty fields beyond cardiology, such as technology-based healthcare; and more broadly clinical research and study designs^31–34^. The two subspecialty journals most frequently publishing cardiac rehabilitation research, the *Journal of Cardiopulmonary Rehabilitation and Prevention* and the *European Journal of Preventive Cardiology*, underwent name changes in 1981 and 2012, respectively, reflecting the expanding focus areas within this field of research^35,36^. The journals most frequently publishing cardiac rehabilitation research are in the subspecialty field of cardiology, followed by rehabilitation or physiatry, and more recently, nursing. These consistent contributions from cardiology journals, combined with the increased presence in allied health and nursing journals, confirm the growing involvement of multidisciplinary professionals in accommodating the delivery of not only exercise training but also education and psychosocial support in cardiac rehabilitation^6^. This trend is further emphasized by the citation metric among the most cardiac rehabilitation-published journals in each research field category. Notably, journals in the subspecialty field of nursing recorded relatively higher citation impact compared to the subspecialty field of rehabilitation. This discrepancy may reflect the broader and interdisciplinary appeal of nursing journals compared to rehabilitation journals. It also aligns with a broader shift in clinical practice from acute, hospital-based care to long-term, outpatient, and community-based models of secondary prevention.

Despite the century-long publication history of cardiac rehabilitation research, over half of the publications from corresponding authors in middle-income countries have encouragingly appeared within the past 5 years. This surge may be attributed to increased investment in cardiac rehabilitation infrastructure, research initiatives, and capacity-building efforts^37^. However, this number may still underestimate the true contributions from middle-income countries, since ~58% of their publications were found in non-indexed local repositories or websites^38^. Additionally, publishing in open-access journals often incurs publication fees, which can be prohibitive for authors in resource-constrained settings^39^. These publication and citation disparities have again been observed across subspecialty fields within cardiology, such as CVD risk factors, and more broadly, cardiology and clinical research^33,34,40^. Strategies to address these disparities could include supporting the indexing of local repositories from lower-income countries, waiving or subsidizing publication fees, and providing training in scientific writing, although its long-term impact of these efforts remains unclear^41^. Strengthening equitable authorship and global collaborations may increase the relevance of findings and support innovations that are locally responsive yet globally scalable.

Cardiac rehabilitation research is anticipated to continue evolving in response to the changing landscape of CVD, adapting to health system characteristics, financial realities, and patient needs^5,6^. Recent publications have highlighted the rising integration of technology-based models or digital platforms, including artificial intelligence, in CVD care—a trend accelerated by the COVID-19 pandemic^17,42^. This may reflect the increasing focus on improving patient access, enabling flexible delivery, and developing scalable, sustainable interventions to reduce disparities in access to secondary prevention. Despite these advancements, research remains comparatively focused on exercise, with less attention directed towards addressing mental health, comorbidities, sub-groups such as the elderly or women, and outcomes such as cardioprotective medication adherence or patient satisfaction. The ongoing focus on exercise in cardiac rehabilitation research is consistent with its central role in program delivery and its strong supporting evidence. However, the predominance of efficacy-focused randomized controlled trials suggests a lack of implementation and real-world studies, which may better inform policy and practice in diverse settings. Incorporating alternative research designs, such as implementation science, and involving patients in program design may enhance the scalability, sustainability, and relevance of cardiac rehabilitation interventions.

The main citation cluster comprising of North America, the United Kingdom, Australia and China, along with a second citation cluster of several European countries, likely reflect shared approaches to cardiac rehabilitation, geographic proximity, and the influence of the American Heart Association, the American College of Cardiology and the European Society of Cardiology in facilitating cross-continent research networks^43–46^. However, disparities in the number and citations of publications are evident, with limited representation from lower-income countries where the CVD burden continues to worsen, aside from notable representations from China and Brazil^4^. The particularly low representation from the African region may be attributed to its lowest availability and density of cardiac rehabilitation among all global regions^47–49^. Recent efforts in the African region to develop cardiac rehabilitation have been commendable and promising^50–52^. Indeed, greater representation both in collaboration or co-authorship with lower-income countries could drive innovations, cost-effective solutions to improve participation rates, and modernize cardiac rehabilitation practices^53–56^. For example, in the field of physical disability rehabilitation, community-based rehabilitation programs developed in lower-income countries have been successfully adapted for remote or underserved areas in high-income countries, such as Australia and Canada^57–60^.

The current bibliometric review has several limitations. Bibliometric reviews primarily involve quantitative analyses, which may overlook qualitative developments in cardiac rehabilitation research. First, the sole use of the Web of Science Core Collection may have excluded regional-specific, country-specific, or non-English publications and not-yet-indexed journals, which may limit the generalizability of the findings, particularly to lower-income countries. Second, the analysis of specific study designs was limited, as these details are not included in its bibliometric records. Regardless, the Web of Science Core Collection remains an appropriate and reliable source, given its broad representation of journals, longest continuous coverage of publications, consistent data on author affiliation, and stable tracking of citations, supporting the reproducibility of the current bibliometric review. Third, changes in journal impact factor, journal subspecialty FWCI, and H-index over time were not accounted for. Fourth, certain types of publication (i.e., book, case report, conference abstract, correction, dissertation or thesis, pre-print, or retracted publication) were excluded, which may have further limited the scope of the current review. To more comprehensively capture the full publication history and changing trends in citations, future reviews could expand their scope to include gray literature, which may uncover economic or political contexts that have shaped the evolution in cardiac rehabilitation research and practice, or to explore the change in journal impact factor, journal subspecialty FWCI, and H-index over time. Additionally, future reviews could use alternative techniques such as text-mining to help reduce the dominance of commonly used author-assigned keywords and analyze more accurately each publication’s contribution to the field of cardiac rehabilitation research.

Modernizing cardiac rehabilitation will require research to more consistently focus beyond exercise and include other patient risk factors, comorbidities, and outcomes. Expanding the use of implementation-focused study designs may help ensure that cardiac rehabilitation remains responsive, relevant, and adaptable to the persistent global burden of CVD and competing demands on health resources.

## Methods

Bibliometric review involves quantitative methods to analyze academic research and literature, exploring publication history, trends in publication keywords, and citations^61^. This study design uses metrics such as journal rankings, country, author contributions, publication keywords, and citation data^61^. The findings are reported in accordance with the preliminary guideline for reporting bibliometric reviews of the bibliometric literature (BIBLIO)^61^.

### Publication search and selection

Cardiac rehabilitation research was identified through a systematic search of the Web of Science Core Collection database for publications in English language up to August 12th, 2024, as specified in the publication. The search targeted titles (field tag: TI) or author keywords (field tag: AK) containing the terms “cardiac rehabilitation” or “heart rehabilitation”. Variation of these terms was included using asterisks (*) and the Boolean operator OR. The Web of Science Core Collection was selected as it provides comprehensive journal-level metadata, standardized author affiliations data, and structured citation metadata within a single database, enabling reproducible evaluation of the trends in journal publications, author collaborations, and citation patterns, which are not collectively available across other databases^62–65^.

Bibliographic data was exported in Excel and text formats, including year and type of publication. This review only analyzed full original research articles, reviews, editorial materials, and letters as categorized in Web of Science, and excluded other types of publication (i.e., book, case report, conference abstract, correction, dissertation or thesis, pre-print or retracted publication). Editorial materials comprise commentaries, discussions, interviews, or symposia between individuals, groups, or organizations^66^. Letters comprise readers’ comments, or questions and answers regarding a publication, addressed to the journal editor^66^. Journals, number of publications, affiliations, and countries of corresponding and co-authors were extracted, as well as author-assigned publication keywords and number of citations. The search strategy, excluded records, included studies, and the list of exported bibliographic data are provided in Supplementary Fig. 1. The country of the corresponding author was categorized according to the World Bank income classifications (i.e., low, lower-middle, upper-middle, or high-income)^67^.

### Publication history: Journal field and quality

To summarize publication history, the total number of journals publishing cardiac rehabilitation research was calculated, and journals were ranked by their total number of cardiac rehabilitation publications. Journal impact factor was obtained from the 2023 Journal Citation Reports by Clarivate^68^; this is calculated by dividing annual citations by the annual number of publications per journal^68^. The median (IQR) impact factors were calculated. The calculations were performed using the bibliometrix package in RStudio version 2024.04.2 +764^69^. The country of origin of journals was obtained from the SCImago Journal & Country Rank and categorized according to the World Bank income classifications^67,70^. Thematic analysis of journal titles was conducted to classify the journals by research field. This analysis was conducted by two independent reviewers (D.M. and K.H.), with any disagreement resolved through discussion. The annual number and citations of publications in journals, each with at least 50 cardiac rehabilitation publications, ranked by the journal’s research field category and number of publications, were visualized using the ggplot2 package in RStudio 2024.04.2 +764^71^. The 2023 FWCI and H-index of the journals most frequently publishing cardiac rehabilitation research in each research field category were compared across field categories. Journal FWCI values were obtained from SciVal; this is calculated by comparing the total citations of all publications in a journal to the average citations of similar publications across all journals^72^. H-index values were obtained from SCImago Journal Rank; this is calculated as the number of publications in a journal that have each received at least the same number of citations as the number of publications^70^.

### Publication history: Publication numbers, country, and author collaboration

The total number, types, and annual number of cardiac rehabilitation publications were aggregated and visualized. The total number of authors, affiliations or institutions, and countries was calculated per publication and aggregated by year. The median (IQR) number of authors per publication and the percentage of publications involving multi-national collaboration were calculated. The distribution of publications across corresponding authors’ country and the income classification of country were aggregated, compared, and visualized.

### Publication keyword

To summarize publication keywords, this part of the review only analyzed cardiac rehabilitation publications from 1990 onwards, the earliest year when author-assigned keywords were available. After variations in spelling and phrasing of keywords were homogenized, the total number of unique keywords was calculated. These were aggregated by 5-year periods; for each, the total number of publications using each keyword was calculated, and the 15 most frequently used keywords in that period were identified. Keywords used in fewer than three publications were excluded from the analysis, while any keyword that was used as frequently as the 15th keyword was also included. Thematic keyword analysis was conducted to create semantically related groupings. This analysis was conducted by two independent reviewers (D.M. and K.H.), with any disagreement resolved through discussion. The identified keywords, ranked by the percentage of publications using each and their thematic group, were compared across the periods and visualized using the ggplot2 package in RStudio 2024.04.2 +764^71^.

### Citations

To summarize citations, the total number and median (IQR) citations of cardiac rehabilitation publications were calculated. The citation patterns across the country and the income classification of the country of the corresponding author were aggregated and compared. The calculations were performed using the bibliometrix package and visualizations using the ggplot2 package in RStudio version 2024.04.2 +764^69^. Citation links and clusters between the most common 10 countries of publication were calculated and visualized using VOSviewer version 1.6.20. A citation link was established between two countries when one country cites the other, regardless of the direction of citation^73^. Citation clusters were formed by grouping countries with similar patterns of citation links^73^.

## Supplementary information

Supplementary Information

## Data Availability

The datasets generated and analyzed during the current study are not publicly available due to licensing restrictions of the Web of Science Core Collection, but are available from the corresponding author on reasonable request.

## Abbreviations

CR: Cardiac rehabilitation
CVD: Cardiovascular disease
IQR: Interquartile range
FWCI: Field-weighted citation impact
H-index: Hirsch index

## Code availability

The underlying code for this study is not publicly available but may be made available to qualified researchers on reasonable request from the corresponding author. Analyses were conducted using RStudio (version 2024.04.2 +764) with the bibliometrix and ggplot2 packages in R, and VOSviewer (version 1.6.20).
