# Supplementary material for: A century of cardiac rehabilitation research: Bibliometric review of publication history, keyword trends, and citations
Source: NPJ Cardiovasc Health. 2025 Jun 26;2:26. doi: 10.1038/s44325-025-00062-w (PMC12912429; doi:10.1038/s44325-025-00062-w)

Supplementary Figure 1. Search strategy, excluded records, included studies, and variables extracted for bibliometric analysis of cardiac rehabilitation publications

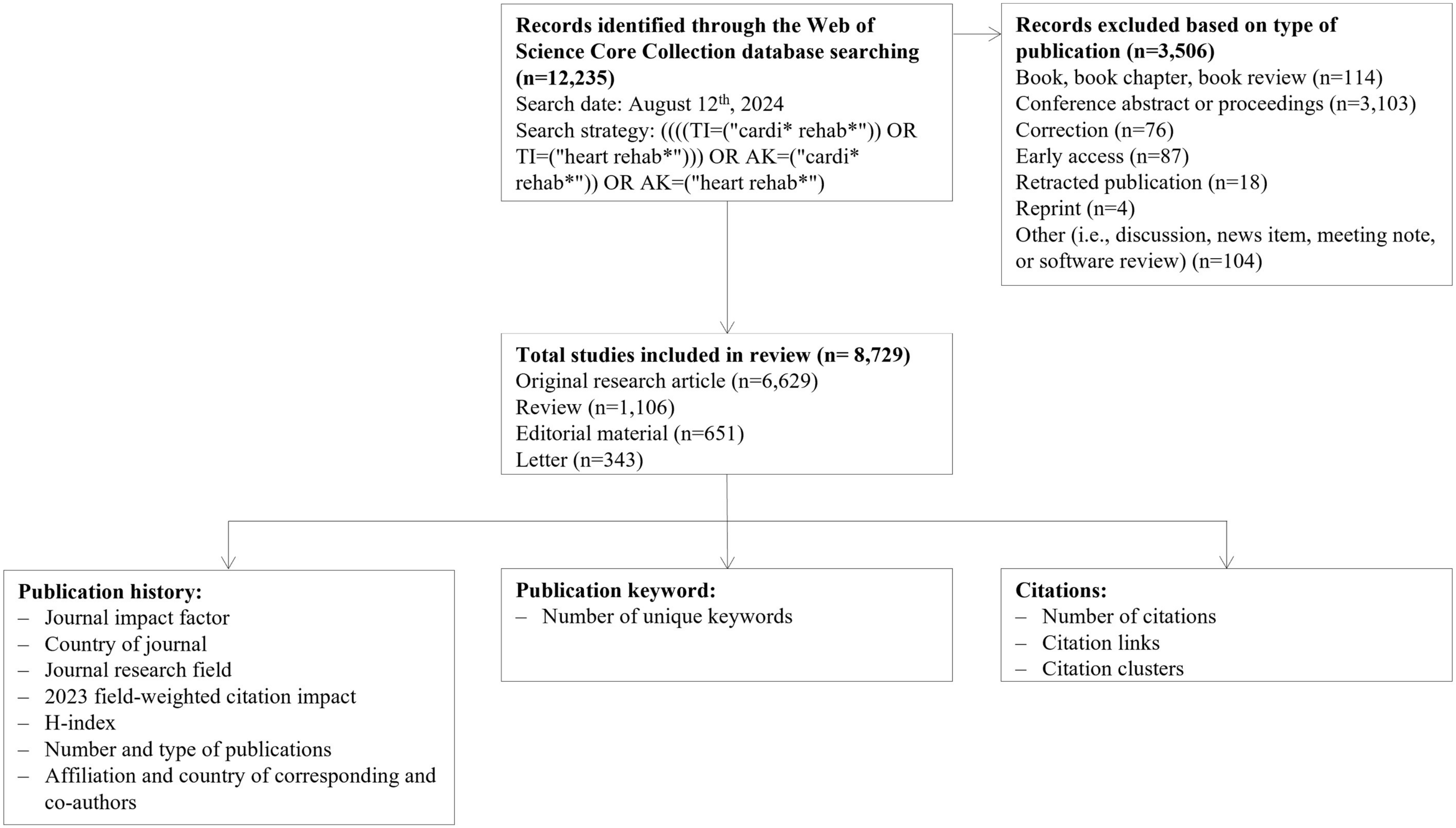

Supplement: Supplementary file 1 — Supplementary Information [file 44325_2025_62_MOESM1_ESM.pdf]
